# Poly r(C) binding protein (PCBP) 1 expression is regulated by the E3 ligase UBE4A in thyroid carcinoma

**DOI:** 10.1042/BSR20170114_RET

**Published:** 2026-04-10

**Authors:** Ming-Peng Zhang, Zaozhi Song, Wei-San Zhang, Jin Tan, Ming-Hui Zhao, Lin-Juan Lian, Jie Cai

**Affiliations:** Department of Geriatrics, Tianjin Medical University General Hospital, Tianjin Geriatrics Institute, Tianjin 300052, China

**Keywords:** E3 Ligase UBE4, Poly r(C) binding protein (PCBP) 1, Thyroid Carcinoma

This article is being retracted from *Bioscience Reports* at the request of the Editor-in-Chief and the Editorial Board. This follows the receipt of a notification from a reader, alerting the Editorial Board to similarities that occur between the western blots of Figure 3A of this article and those published in multiple other papers by different authors. Specifically:

- The UBE4A bands also appear in the following publications:
- Li et al. 2020 (https://doi.org/10.1186/s12935-020-01441-2) as the Figure 4a SNAIL1/Kyse150 bands
- Zhou et al. 2020 (https://doi.org/10.3389/fphar.2020.602307) as the Figure 4a elF2a bands
- The GAPDH bands also appear in the following publications:
- Li et al. 2020 (https://doi.org/10.1186/s12935-020-01441-2) as the Figure 4a GADPH bands
- Yin et al. 2020 (https://doi.org/10.3892/ol.2020.11783) as the Figure 2a RBM38 bands.

The authors have been contacted with regard to the retraction and have not responded to the Journal's queries or the concerns raised. Given the extent of the issues raised, the Editorial Board stands by the decision to retract the article.

